# Docosahexaenoic Acid Levels in Blood and Metabolic Syndrome in Obese Children: Is There a Link?

**DOI:** 10.3390/ijms160819989

**Published:** 2015-08-21

**Authors:** Carlotta Lassandro, Giuseppe Banderali, Giovanni Radaelli, Elisa Borghi, Francesca Moretti, Elvira Verduci

**Affiliations:** 1 Department of Pediatrics, San Paolo Hospital, Department of Health Science, University of Milan, I-20142 Milan, Italy; E-Mails: carlotta.lassandro@unimi.it (C.L.); giuseppe.banderali@unimi.it (G.B.); giovanni.radaelli@unimi.it (G.R.); francesca.moretti@unimi.it (F.M.); 2 Microbiology Unit, Department of Health Sciences, University of Milan, I-20142 Milan, Italy; E-Mail: elisa.borghi@unimi.it

**Keywords:** metabolic syndrome, obesity, DHA, n-3 LCPUFA, glucose metabolism, lipid profile, blood pressure, NAFLD

## Abstract

Prevalence of metabolic syndrome is increasing in the pediatric population. Considering the different existing criteria to define metabolic syndrome, the use of the International Diabetes Federation (IDF) criteria has been suggested in children. Docosahexaenoic acid (DHA) has been associated with beneficial effects on health. The evidence about the relationship of DHA status in blood and components of the metabolic syndrome is unclear. This review discusses the possible association between DHA content in plasma and erythrocytes and components of the metabolic syndrome included in the IDF criteria (obesity, alteration of glucose metabolism, blood lipid profile, and blood pressure) and non-alcoholic fatty liver disease in obese children. The current evidence is inconsistent and no definitive conclusion can be drawn in the pediatric population. Well-designed longitudinal and powered trials need to clarify the possible association between blood DHA status and metabolic syndrome.

## 1. Introduction

Childhood obesity is one of the most pressing public health issues [1] and a major risk for adult obesity and related comorbidities [2], which may already develop during pediatric age, such as insulin resistance, non-alcoholic fatty liver disease (NAFLD), type-2 diabetes mellitus (T2DM), dyslipidemia, hypertension, metabolic syndrome (MS), obstructive sleep apnea, joint problems, gallstones, and psychosocial problems [3].

There has been a marked increase in the prevalence of both obesity and metabolic syndrome in children over the past decades [4]. Metabolic syndrome is defined as a cluster of cardiovascular and type-2 diabetes risk factors, as hypertension, altered glucose metabolism, dyslipidemia, and abdominal obesity [5]. More than 40 definitions have been proposed to define metabolic syndrome and none of these is “universally accepted” [6]. However, some common features include the assessment of obesity (usually through body mass index (BMI) or waist circumference), the measurement of blood pressure and blood lipids (usually triglycerides (TG), high-density lipoprotein (HDL) cholesterol or low-density lipoprotein (LDL) cholesterol), and evaluation of risk factors associated with diabetes (fasting glucose, glucose tolerance and insulin resistance) [6].

Among different criteria to define metabolic syndrome in children, International Diabetes Federation (IDF), National Cholesterol Education Program’s Adult Treatment Panel III (ATP), and World Health Organization (WHO) criteria (with or without modification) are the most used [7]. Although there is no agreement on what criteria to use, recently the use of IDF-based criteria has been suggested [7]. The IDF definition of metabolic syndrome [8] in childhood is different according to age (6 to <10, 10 to <16, and ≥16 years). Below the age of 10 years the metabolic syndrome is not diagnosed; from 10 to 16 years the diagnosis requires the presence of abdominal obesity (waist circumference greater than or equal to the 90th percentile) plus the presence of two or more of: blood level of triglycerides ≥150 mg/dL, level of HDL-cholesterol <40 mg/dL, systolic blood pressure ≥130 or diastolic blood pressure ≥85 mmHg, and fasting plasma glucose ≥100 mg/dL or known T2DM. In adolescents aged ≥16 years the IDF adult criteria can be used [8].

The prevalence of metabolic syndrome in children is highly variable according to the criteria used. A systematic review of studies using one of the main three criteria (IDF, ATP III, WHO) to define MS [7], highlighted that the median prevalence of metabolic syndrome was 3.3% in the whole population of children while it was 11.9% and 29.2% in overweight and obese children, respectively. Moreover, the median metabolic syndrome prevalence was higher in older (5.6%) than younger children (2.9%) and was higher in boys (5.2%) than girls (3.1%) [7]. Similar results were published in National Health and Examination Survey (NHANES) report [9] showing a metabolic syndrome prevalence in adolescents of 7.9% in boys compared to 6.7% in girls. Moreover, the prevalence of metabolic syndrome tends to be higher in pubertal than prepubertal children [6].

Finally, the link between metabolic syndrome and NAFLD should be evaluated. Indeed, in parallel to the rising epidemic of metabolic syndrome, also the prevalence of NAFLD has increased [10]. NAFLD is also often associated with clinical and biochemical features of metabolic syndrome [11] in children [12]. Although NAFLD might be considered the liver manifestation of the metabolic syndrome, this concept may be outdated [11]. A vicious cycle between NAFLD and metabolic syndrome could exist in pediatric age [13]. A recent review suggested that NAFLD is a determinant for the onset of the metabolic syndrome and, therefore, a precursor [11]. Therefore, although to date NAFLD is not a component of the diagnostic criteria for metabolic syndrome its importance needs to be stressed [11,14].

## 2. Docosahexaenoic Acid: Metabolism and Properties

Long chain n-3 polyunsaturated fatty acids (n-3 LCPUFAs) are long chain fatty acids (20 carbons or more), with the first double bond located after the third carbon from the methyl end [15]. Together with eicosapentaenoic acid (C20:5 (n-3), EPA) and docosapentaenoic acid (C22:5 (n-3), DPA), docosahexaenoic acid (C22:6 (n-3), DHA) is a main n-3 LCPUFAs in food sources [16]. Alfa-linolenic acid (C18:3 (n-3), ALA), an essential dietary fatty acid that cannot be synthesized in humans, is the precursor of all n-3 LCPUFAs [4]. Humans can convert ALA to EPA and DHA, but, since conversion efficiency is low, an adequate dietary intake is required [16].

Significant amounts of EPA and DHA characterize fish and derivative fish oil, especially salmon, tuna, mackerel, anchovy, and sardines, while ALA can be found in vegetable oils [4]. Moreover, DHA is found in human milk and it is necessary for optimal development of the brain and the retina of the infant [16]. However, DHA content in human milk varies substantially depending on the maternal intake of DHA, genetics, and other environmental factors [17], such as maternal smoking during pregnancy [18].

EPA and DHA intake through the diet increases the n-3 LCPUFA content of phospholipids, the main component of the cell membrane, also reducing arachidonic acid (AA) levels [15]. Fatty acids in erythrocyte are considered as the most reliable markers of habitual dietary intake of n-3 LCPUFAs, reflecting the intake over several months [19]. Fatty acids in plasma phospholipids reflect the intake over a relatively short period [19]. However, plasma phospholipids may reflect the fatty acids composition of erythrocyte lipids [20] and, in turn, erythrocyte fatty acids composition may reflect the fatty acids (especially PUFAs) composition of muscle membrane phospholipids [21].

n-3 LCPUFAs are associated with health benefits. EPA and DHA are essential for optimal fetal development and healthy aging [22], constitute phospholipids of most biological membranes with a relevant role in structure and function [16], have anti-inflammatory properties and modulate viscosity of cell membranes [22], and contribute to membrane fluidity, which can influence the function of membrane receptors [16]. Moreover, EPA and DHA are the precursors of numerous metabolites that function as lipid mediators with a plausible beneficial role in the prevention or treatment of several diseases [22]. Series D resolvins and protectins, two active metabolites derived from DHA, may modulate the inflammatory response by decreasing cytokine production and promoting the resolution of inflammation [23]. These metabolites could have a potential and important role in metabolic syndrome since a low-grade inflammation characterizes this condition [24]. It has been suggested that reducing the ratio of n-6/n-3 PUFA in diet (currently estimated about 10:1 in the Western diet), the risk factors of metabolic syndrome could be reduced [25]. However, the evidence about the relationship of n-3 PUFAs and components of the metabolic syndrome is inconsistent [26].

### Topic of Review

This paper reviews the literature published in the last decade and discusses the relationships of blood DHA with each component of IDF criteria for metabolic syndrome (obesity, alteration of glucose metabolism, blood lipid profile and blood pressure) and NAFLD in obese children.

## 3. Childhood Obesity: Relationship between DHA Content in Plasma and in Erythrocytes and Metabolic Syndrome Criteria and NAFLD

### 3.1. Obesity

Abdominal obesity, rather than obesity, can predict the presence of insulin resistance and related metabolic syndrome [27]. A systematic review and meta-analysis [28], including a total of 21 studies (11 conducted in childhood) for a total of 1575 participants, was performed in order to evaluate LCPUFA status in blood in overweight/obese subjects. Compared with healthy controls, overweight/obese subjects showed lower DHA levels in total plasma lipids but no difference was found in plasma phospholipid and plasma cholesteryl ester fraction, suggesting that DHA deficiency might be not systemic [28].

Only a few studies have been conducted considering DHA status in blood among the obese pediatric population [29,30,31,32,33,34,35,36]. A case-control study on 67 normolipidemic obese children, aged 8–12 years, and 67 age- and sex-matched normal-weight children, observed that obese children showed significantly lower levels of DHA/ALA ratio in total plasma fatty acids compared to normal-weight controls [29]. Moreover obese children in the highest quartile of BMI z-score showed lower levels of DHA, DHA/AA, and DHA/ALA ratios than normal-weight children, despite a higher dietary PUFAs intake, suggesting a metabolic dysfunction in the synthetic pathway of the n-3 series in severely obese children [29]. Saito *et al.* [30] assessed the analysis of fatty acid composition of plasma phospholipids in 32 obese children and found an inverse association (almost statistically significant) of DHA content with BMI. Similarly, a study found that 60 overweight adolescents had lower total n-3 PUFA and DHA concentrations in plasma phospholipids, compared to normal-weight controls [31]. Another study conducted on adolescents, showed that obese girls, but not boys, had lower concentrations of n-3 PUFAs, including DHA in plasma phospholipids compared to normal-weight controls, and that DHA was inversely associated with all fat depots, measured by magnetic resonance imaging, except visceral adipose tissue, both in girls and in boys [32]. Furthermore, in another study, obese children showed after one year of nutritional-behavioral intervention a decreased BMI z-score of 12.3% and increased plasma levels of DHA and DHA⁄AA ratio, compared to baseline, with a consequent disappearance of the difference for DHA⁄AA ratio between obese children and normal-weight controls [33]. It should be interestingly noted that in this study, whereas the plasma PUFA increased after one year, the dietary PUFA intake decreased [33]. However, further studies are needed to better clarify the role of dietary change on specific plasma fatty acid in obese children. On the contrary, a study performed on obese prepubertal children with metabolic syndrome showed higher levels of DHA in total plasma lipids, compared to normal-weight controls while no difference was observed in plasma phospholipid and plasma cholesteryl ester fraction [34]. Another study found no difference in total plasma lipid levels of DHA between obese children and normal-weight controls [35].

Moreover, in a recent study, 33% of obese children showed an n-3 index (calculated by adding EPA% and DHA% (weight/weight) values) <4.0 (associated to high risk of cardiovascular disease) in erythrocytes compared to 17% of non-obese children, suggesting that obese children may have an altered erythrocyte fatty acid composition [36].

As a whole, several discussed studies found blood DHA may be lower in obese children and negatively associated with the degree of obesity, but further studies are needed to better understand the relationship between DHA status and obesity.

### 3.2. Glucose Metabolism Alterations

An important key factor in the pathogenesis of metabolic syndrome is insulin resistance [37], a whole-body decrease in the ability of insulin to stimulate the use of glucose by muscles and adipose tissue and to suppress glucose production in the liver [38]. Prevalence of insulin resistance has increased significantly in children in the last three decades [39]. Indeed, the analysis of the US NHANES 1999–2002, involving 1802 adolescents without diabetes, has shown that insulin resistance prevalence was 52% among obese children [40]. A marked increase of the prevalence of pre-diabetic stages’ conditions and type 2 diabetes mellitus among obese children and adolescents has also been observed [39].

Low levels of LCPUFAs, especially DHA, and a high n-6/n-3 LCPUFA ratio in skeletal muscle membrane phospholipids have been associated with insulin resistance in adults [41]. Moreover, membrane flexibility, determined by the polyunsaturated fatty acid/saturated fatty acid (PUFA/SFA) ratio, could impact on the effectiveness of glucose transport by insulin-independent glucose transporters (GLUTs) and the insulin-dependent GLUT4 [42].

Literature concerning blood DHA status in the pediatric obese population is scanty [30,31,32,33,43,44]. In obese children, DHA content in plasma phospholipids was not associated with parameters of glucose metabolism as fasting glucose, fasting insulin, and homeostasis model assessment-insulin resistance (HOMA-IR) [30]. The lack of association between plasma DHA levels and HOMA-IR was confirmed in other different studies [31,33]. On the contrary, a study found that DHA in plasma phospholipids was inversely associated with serum insulin and HOMA β-cell function [32] and other studies conducted on obese children showed that HOMA-IR was negatively associated with DHA in plasma phospholipids [43,44].

On the whole, the evidence from existing literature is not conclusive about the association between DHA status in blood and glucose metabolism alterations in obese children. However, it should be pointed out that breastfeeding, as the best feeding practice in early life, could have a protective role on glucose metabolism derangements [45,46], possibly also involving DHA in breast milk [18,45]. Indeed, fatty acids composition of breast milk, including DHA, may increase LCPUFAs in skeletal muscle membranes protecting against insulin resistance, β-cell failure, and type-2 diabetes [18,45].

### 3.3. Abnormal Blood Lipid Profile

The alterations of blood lipid profile associated with metabolic syndrome are usually characterized by increased triglycerides, very-low-density lipoproteins (VLDLs), small dense LDL particles, and reduced HDL cholesterol levels [47,48]. Visceral obesity and insulin resistance could be key factors involved in the promotion of atherogenic dyslipidemia by increasing the synthesis of TG-rich VLDLs in the liver [4].

In adults, increased plasma levels of EPA and DHA might be inversely associated with the risk of the progression of coronary atherosclerosis, sudden cardiac death, and coronary heart disease, clinical conditions related to risk factors for cardiovascular disease, including dyslipidemia [49].

The possible relationships of DHA with blood lipid profile have been poorly investigated in the pediatric population [30,31,33]. A study performed on 32 obese children showed that plasma phospholipids’ DHA content was negatively associated with VLDL-triglyceride, a major factor involved in the development of metabolic syndrome [30]. A cross-sectional study did not find any associations of DHA in both plasma phospholipids and cholesteryl esters with parameters of blood lipid profile in overweight adolescents, while the PUFA/SFA and linoleic acid levels in plasma phospholipids were positively associated with HDL cholesterol [31]. Another study, analyzing total plasma fatty acids on 57 normolipidemic obese children, concluded that after one year of nutritional-behavioral intervention changes in plasma DHA and DHA/AA ratio (both increased) were inversely associated with changes in plasma total TGs [33].

In conclusion, association between plasma DHA levels and blood lipid profile alterations in pediatric obese population is inconsistent.

### 3.4. Blood Pressure Alterations

The prevalence rates of hypertension and obesity are increasing worldwide in children [50]. The blood pressure lowering effect of DHA, observed in adults, could be mediated by the adenosine triphosphate (ATP) release from the endothelium, which increases vasodilation by stimulating the release of nitric oxide, and by the decrease in noradrenaline levels [51].

To our knowledge, only one study evaluated the association between DHA status in blood and blood pressure in obese children. This study, analyzing plasma fatty acid composition in 60 overweight adolescents found that DHA status was not associated with systolic blood pressure [31].

Regarding breastfeeding, a systematic review stated that breastfeeding has a small protective effect against high systolic blood pressure, although residual confounders had to be eliminated [45]. One of the plausible mechanisms that has been suggested to explain this protective effect is represented by the presence of LCPUFAs, including DHA, which are important structural components of the vascular endothelium [45]. In a multicenter, randomized, controlled trial, children fed with a formula supplemented with LCPUFAs (mainly DHA and EPA) showed at age 6 years lower blood pressure than children fed with a formula without LCPUFAs [52].

In conclusion, while in adults an association of DHA status with blood pressure has been observed, in obese children the literature is limited and further longitudinal studies would be desirable.

### 3.5. NAFLD (Non-Alcoholic Fatty Liver Disease)

In children of industrialized countries, NAFLD is the most common chronic liver disease, reaching a prevalence up to 80% in obese or overweight children [53]. NAFLD includes different diseases ranging from “simple” liver steatosis, with pathological accumulation of fat in excess of 5% of liver weight, non-alcoholic steatohepatitis (NASH), with different degree of inflammation and fibrosis, to end-stage liver disease with cirrhosis and hepatocellular carcinoma [54].

Obese adults with NAFLD showed lower levels of n-3 LCPUFAs, EPA, DHA, and a higher n-6/n-3 ratio in liver than controls [55]. Lower n-3 LCPUFA levels in liver have also been associated with lower levels in erythrocyte phospholipids [56]. The low n-3 LCPUFA levels in liver, by promoting the synthesis of fatty acids and triglycerides with parallel imbalance in the oxidation of fatty acids and export of triglycerides from the liver, could determine fat accumulation and promote liver steatosis [55,57]. To our knowledge there are no studies investigating the association between fatty acids composition of liver phospholipids, and especially liver levels of DHA, and NAFLD in obese children. Only one study showed that in obese children with single-nucleotide polymorphism (SNP), 276G>T at adiponectin gene, the increased liver echogenicity could be associated with higher levels of n-6 PUFA in plasma phospholipids (unpublished results, presented at 44th ESPGHAN Annual Meeting, Sorrento) [58]. However, some trials evaluated the effect of DHA supplementation on pediatric NAFLD [59,60]. A reduced liver hyperechogenicity was observed in children with NAFLD after DHA supplementation for 6, 12, 18, and 24 months [59]. After 18 months of DHA treatment an improvement of histo-pathological parameters (NAFLD activity score, ballooning, and steatosis) has been also observed [60].

Only one study has evaluated the association between breastfeeding and NAFLD in children. This retrospective study suggested that breastfeeding might be protective against NASH and liver fibrosis, suggesting a long-lasting effect of breast milk DHA [61]. The authors speculated that DHA, supplied by breast milk, could be protective, acting as a peroxisome proliferator-activated receptors (PPAR)-agonist, a transcription factor involved in protection against fibrosis [61,62].

In conclusion, further studies are needed to evaluate the existence of a relationship between DHA status in blood and NAFLD in children and to confirm the protective role of DHA in breast milk against NAFLD progression.

## 4. Discussion and Conclusion

The metabolic syndrome, considered in the past as an adulthood disorder, also affects children with increasing prevalence [4,5].

DHA has been associated with beneficial effects on health and in treatment of several diseases [22], such as cardiovascular disease, cancer, inflammatory, thrombotic and autoimmune disease, coronary heart disease, hypertension, and type-2 diabetes, in adults [16]. The reduction of dietary n-6/n-3 PUFA ratio could reduce risk factors associated with metabolic syndrome [25,63].

Table 1 summarizes the observed relationship between DHA content in plasma and erythrocytes and components of IDF criteria for metabolic syndrome in obese children. The current evidence is inconsistent and no definitive conclusion can be drawn in the pediatric population. Further well-designed studies are needed to evaluate a possible role of DHA supplementation as a prevention strategy of obesity-related comorbidities in childhood.

**Table 1:** DHA status in blood and components of IDF criteria for metabolic syndrome in obese children.

Metabolic Syndrome Components [Ref]	Blood DHA Status
Obesity [29,30,31,32,33,34,35,36]	DHA content is lower in obese children and negatively associated with the degree of obesity, except for two studies [34,35]
Glucose metabolism alterations [30,31,32,33,43,44]	Inconsistent results
Abnormal blood lipid profile [30,31,33]	Inconsistent results
Blood pressure alterations [31]	None association with systolic blood pressure
